# 

**Published:** 1887-07

**Authors:** 


					﻿Vol VIk
JULY, 1887.
NO. 7.
THE
HOMEOPATHIC DHY^lClAp,
A MONTHLY JOURNAL OF MEDICAL SCIENCE.
** He u a freeman whom truth makes free, and all are slaves beside."
“Seek the Truth: come whence it may, cost what it will."
EDITED BY
Edmund j. lee, m. d.’
AND
WALTER XI. JAMES, XI. D.
PHILADELPHIA:
No. 1183 SPRUCE STREET.
3ST 33 W TT'O 2B 3C
A. L. CHATTERTON & COMPANY, Publishbb’S Aoknt&
LOWDONi
ALFRED HEATH A CO., Sons Agents bob Gbeat Britain,
114 Ebury Street, Eaton Square.
Yearly Subscription, $2.30, Invariably in advance. Single numbers, 23 cts.
Entered at the Philadelphia Peet-Office aa Second-Claw Mall Matter.
				

